# Global research trends on thyroid hormones and neurodegenerative diseases: a bibliometric study from 2015 to 2025

**DOI:** 10.3389/fnagi.2026.1780027

**Published:** 2026-04-02

**Authors:** Yan Lu, Xiaodong Zhang, Lanting Zhou, Fan Ye

**Affiliations:** 1 Department of Medical, School of Medicine, Wuhan University of Science and Technology, Wuhan, China; 2 Department of Anesthesiology, Xiangyang Central Hospital, Affiliated Hospital of Hubei University of Arts and Science, Xiangyang, China; 3 Institute of Neuroscience and Brain Science, Xiangyang Central Hospital, Affiliated Hospital of Hubei University of Arts and Science, Xiangyang, China

**Keywords:** Alzheimer’s disease, bibliometrics, hypothyroidism, neurodegenerative diseases, thyroid hormones

## Abstract

**Objective:**

Despite extensive research on the role of thyroid hormone (TH) in neurodegenerative diseases (NDs), no bibliometric study has been conducted to evaluate their interrelationship. To fill this gap, this study conducts an analysis of literature published between 2015 and 2025, aiming to uncover research trends and emerging hotspots and to offer valuable guidance for subsequent investigations.

**Methods:**

Publications from 2015 to 2025 were collected from the Web of Science (WoSCC) and Scopus databases and subsequently analyzed with R software, VOSviewer, and CiteSpace.

**Results:**

This study included 820 papers retrieved from the WoSCC database and 2,039 relevant papers retrieved from the Scopus database. The annual publication output in this field increased steadily from 2015 to 2025, reflecting growing scholarly interest. Among all contributing countries, China produced the greatest number of publications, followed successively by the United States, Italy, Japan, and Brazil. Furthermore, China not only led in research productivity but also demonstrated the strongest international collaborative networks. The *Journal of Clinical Endocrinology and Metabolism* had the highest number of publications and citation rate. Comprehensive analysis results indicate that research hotspots in this field mainly focus on the epidemiological evidence of TH secretion abnormalities and NDs, the mechanisms of TH in NDs, and the role of thyroid hormone–mediated remyelination and neural regeneration in diseases such as MS.

**Conclusion:**

In the past decade, research on TH and NDs has attracted increasing global attention, reflecting growing scholarly interest in this field. The present bibliometric analysis systematically identified the key research hotspots and evolving trends of TH in NDs. These findings provide a comprehensive overview of the current research landscape and may serve as a reference framework for future investigations in this area.

## 1 Introduction

Neurodegenerative diseases (NDs) are a heterogeneous group of disorders marked by the progressive impairment and loss of neuronal structure or function affecting the central and peripheral nervous systems. In addition to the most widely recognized forms—Alzheimer’s disease (AD) and Parkinson’s disease (PD)—this group includes Huntington’s disease (HD), amyotrophic lateral sclerosis (ALS), multiple sclerosis (MS), and spinocerebellar ataxia (SCA), among others. Most NDs ultimately culminate in dementia. AD is the most prevalent neurodegenerative disorder and the primary cause of dementia worldwide, responsible for approximately 60–80% of all dementia cases (Erkkinen et al., 2018). By 2050, approximately 150 million people worldwide are expected to be affected. Patients and their families will face not only substantial physical and psychological burdens but also significant economic pressures (Nandi et al., 2022; GBD 2019 Dementia Forecasting Collaborators, 2022). Unfortunately, existing treatment methods can only alleviate symptoms and cannot fundamentally resolve the disease. The efficacy of commonly used clinical drugs gradually diminishes as the disease progresses (Sudhakar and Richardson, 2019), not only due to the complex pathological processes of neurodegenerative diseases but also because the barrier and protective properties of the blood–brain barrier (BBB) prevents most drugs from effectively acting within the nervous system (Sivandzade and Cucullo, 2021). Therefore, exploring new therapeutic strategies is crucial for improving the treatment of neurodegenerative diseases.

Thyroid hormones (TH), namely thyroxine (T4) and triiodothyronine (T3), are crucial growth regulators. Additionally, thyroid stimulating hormone (TSH), free thyroxine (FT4), and free triiodothyronine (FT3) are the fundamental indicators of thyroid function. They not only play a vital role in the development and growth of the central nervous system, but also maintain the integrity of neuronal structure and function in adulthood (Bavarsad et al., 2019). Moreover, the lipophilic nature of thyroid hormone enables it to cross the blood–brain barrier, thereby exerting a protective effect under the pathological conditions of neurodegenerative diseases (Chatzitomaris et al., 2017). Supplementation with T3 has been found to improve depressive-like behaviors in AD and shows potential in delaying disease progression (Maglione et al., 2022a). TH can induce the differentiation of dopaminergic neurons, suggesting a potential link to the treatment of Parkinson’s disease (PD). The level of TH, especially FT3, has been associated with cognitive impairment in PD patients and may represent a promising biomarker indicative of cognitive dysfunction in PD (Peng et al., 2024). Multiple sclerosis (MS) represents an autoimmune condition involving progressive demyelination of the central nervous system, in which oligodendrocytes are essential for myelin generation and repair (Kuhn et al., 2019). TH is an essential factor in myelin formation, and an increase in FT4 levels can affect the maintenance of normal myelin. FT4 may be a risk factor for MS, promoting its occurrence (Ren et al., 2025). Currently, extensive research has focused on elucidating the association between TH and NDs; however, the results remain inconsistent, and the underlying mechanisms are still unclear. Although some studies have shown that TH has beneficial effects on NDs, others have indicated that elevated TH levels may be a potential driving factor in AD and PD, possibly through mechanisms involving neuronal degeneration (Lin et al., 2021). Hence, a systematic evaluation of the research development, key outcomes, and prospective directions of TH in NDs is necessary to provide scholars with an updated overview of the cutting-edge advancements and research hotspots in this area.

Bibliometrics is a method used for the objective and quantitative analysis of published research (Shan et al., 2022; Chen et al., 2024). By quantitatively analyzing factors such as publishing countries, authors, and institutions within relevant domain literature, bibliometrics can efficiently identify research hotspots. Owing to this advantage, it has been widely applied in the medical field. This study carries out a systematic bibliometric analysis of research articles published during the period 2015–2025, focusing on the interactions between TH and NDs, with the aid of R software, VOSviewer, and CiteSpace. By employing bibliometric techniques, the study aims to map research hotspots, delineate emerging trends, and provide theoretical insights to support the potential therapeutic use of TH in neurodegenerative disorders.

## 2 Materials and methods

### 2.1 Data collection

All data analyzed in this study were retrieved from the Web of Science Core Collection (WoSCC; Wuhan University of Science and Technology version) and the Scopus database on September 13, 2025. The search terms used in this study were as follows: (“Neurodegenerative” OR “Neurodegenerative Disorder” OR “Neurologic Degenerative” OR “Alzheimer’s disease” OR “Alzheimer” OR “AD” OR “Parkinson’s disease” OR “PD” OR “Huntington’s disease” OR “HD” OR “Amyotrophic Lateral Sclerosis” OR “ALS” OR “Spinal Cerebellar Ataxia” OR “SCA” OR “Multiple Sclerosis” OR “Cognitive impairment” OR “Cognitive decline”) AND (“Thyroid Hormone*” OR “Thyroid hormones” OR “Thyroxine” OR “Triiodothyronine” OR “Levothyroxine”). The search period covered January 1, 2015, to September 13, 2025, and the language was restricted to English. Only articles and reviews were included (for the detailed search strategy, see Supplementary material 1). A total of 820 papers were retrieved from the WoSCC database and saved as TXT files, while 2039 papers were retrieved from the Scopus database and saved in CSV format, including full records and cited references. After removing duplicates, the WoSCC database retained 820 papers (Repeat *n =* 0), and the Scopus database retained 2039 papers (Repeat *n =* 0). Verification confirmed that there were no duplicate papers in either database.

### 2.2 Data analysis

Given the inconsistent data export formats among databases and the potential data loss after merging, this study obtained plain text data from the WoSCC and Scopus databases and imported them into software for bibliometric analysis. WoSCC is a globally recognized database that provides comprehensive and accurate article coverage. Following a comparison of the two databases, we used the WoSCC as the main data source for analysis, whereas findings derived from Scopus, including publication trends and keyword clustering, are presented in the Supplementary materials. The annual trend of publications was visualized using Origin 2018 software. VOSviewer was employed to construct cooperation networks among countries and institutions, as well as to carry out journal co-citation and keyword co-occurrence analyses. In co-authorship network analysis, we set the following parameters: minimum number of documents of a country ≥ 5; minimum number of documents of an organization ≥ 4. In the co-citation of source analysis, we set the following parameters: minimum number of citations of a source ≥80. Additionally, in the co-occurrence of keyword analysis, the parameters were set as follows: minimum number of occurrences of a keyword ≥7, and we excluded “neurodegenerative diseases,” “Neurodegenerative,” “Neurodegenerative Disorder,” “Thyroid hormones” “Triiodothyronine,” keywords and merge synonymous keywords. VOSviewer employs association strength normalization methoda to construct the co-occurrence network. CiteSpace was used to identify citation bursts, while the bibliometrix package in R was utilized to further achieve structured mapping and visualization of scientific knowledge. This study adopts the classical scientometric method embedded in the bibliometrix package. In the implementation process, the sample data were first divided into several time intervals. Subsequently, research themes for each period were identified based on keyword co-occurrence networks and clustering algorithms. Finally, the inclusion index was calculated to trace the inheritance and evolution of themes along the temporal axis. The impact factors (IFs) of journals were extracted from the Journal Citation Reports (JCR), 2024 edition, to ensure consistency and accuracy in evaluating journal influence. These analytical tools and methods collectively provide a comprehensive understanding of the knowledge structure and research frontiers in this field.

## 3 Results

### 3.1 Publication of the literature dataset and the overall trend

**Figure 1:**
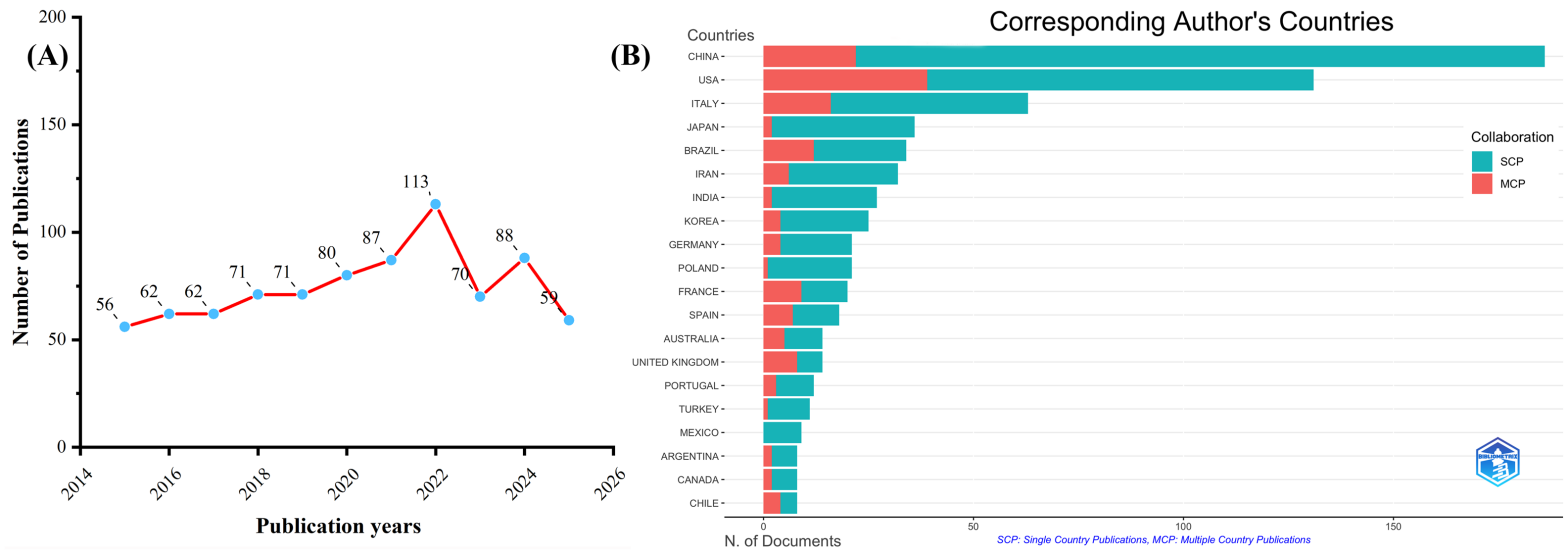
Trends in annual publication outputs: the relationship between TH and NDs from 2015 to 2025. **(A)** Trends of annual publication outputs. **(B)** Distribution of corresponding authors’ countries and cooperation.

After conducting searches based on the specified criteria, 820 documents were retrieved from the WoSCC database and 2,039 documents from the Scopus database. After removing duplicate records, the final analysis dataset was established. The publication output concerning TH and NDs demonstrated a general upward trajectory from 2015 to 2025 (Figure 1A). From 2020 to 2022, the volume of publications rose sharply over the study period and attained its maximum level in 2022. In that year, the WoSCC database recorded 113 publications, while the Scopus database recorded 279 publications (see Supplementary Figure 1). After 2023, the number of publications showed a slight decline, suggesting that research activity in this field rose sharply during the COVID-19 pandemic and stabilized afterward. Although the sample sizes of the two databases differ, analysis of Figure 1 and Supplementary Figure 1 indicates that both present essentially the same publication trends.

**Figure 2:**
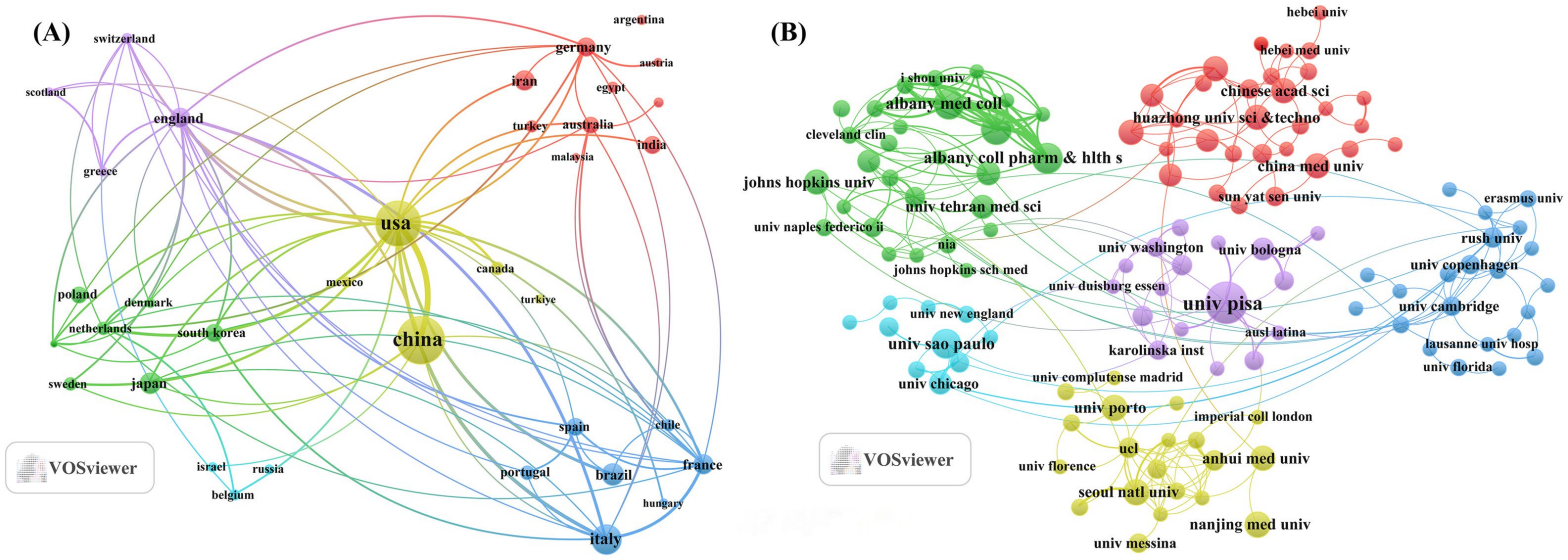
Map of countries/regions and institutions the relationship between TH and NDs from 2015 to 2025. **(A)** Map of cooperation between different countries. **(B)** Map of cooperation between different institutions.

Examination of the corresponding authors’ country distribution showed that China contributed the highest publication output with 186 articles accounting for 22.7% of all documents. The United States (*n =* 131), Italy (*n =* 63), Japan (*n =* 36), and Brazil (*n =* 34), followed closely behind, reflecting the global distribution of research in this field. Among the top five most productive countries, China (11%) and the United States (19.5%) stand out as leading contributors in terms of publication volume and demonstrate robust international collaboration networks, ranking highly in multi-country cooperation (MCP). In contrast, Japan (1%) shows significantly less international collaboration, suggesting that research in this area there is more domestically focused (see Figure 1B; Table 1). The country collaboration network further supports this observation. China and the United States serve as the two major cooperation hubs in this field, indicating that both nations not only attach great importance to research on TH and NDs but also actively promote in-depth international cooperation (see Figure 2A). Although the University of Pisa in Italy (*n =* 25) has the highest number of institutional publications, most of the productive institutions are located in China. Among the top 15 institutions, eight are Chinese, further underscoring China’s central position in this research domain (see Figure 2B; Table 2).

**Table 1:** Most relevant countries by corresponding authors of the relationship between MedDiet and DM.

Country	Articles	Articles %	SCP	MCP	MCP %
China	186	22.7	164	22	11
USA	131	16	92	39	19.5
Italy	63	7.7	47	16	8
Japan	36	4.4	34	2	1
Brazil	34	4.1	22	12	6
Iran	32	3.9	26	6	18.8
India	27	3.3	25	2	7.4
Korea	25	3	21	4	16
Germany	21	2.6	17	4	19
Poland	21	2.6	20	1	4.8
France	20	2.4	11	9	45
Spain	18	2.2	11	7	38.9
Australia	14	1.7	9	5	35.7
United Kingdom	14	1.7	6	8	57.1
Portugal	12	1.5	9	3	25
Turkey	11	1.3	10	1	9.1
Mexico	9	1.1	9	0	0
Argentina	8	1	6	2	25
Canada	8	1	6	2	25
Chile	8	1	4	4	50
Egypt	7	0.9	5	2	28.6
Israel	7	0.9	3	4	57.1
Netherlands	7	0.9	2	5	71.4
Russia	7	0.9	6	1	14.3
Sweden	7	0.9	5	2	28.6

**Table 2:** Most relevant affiliations of the relationship between TH and NDs.

Affiliation	Articles (*n*)
University of Pisa	25
Albany College of Pharmacy and Health Sciences	13
Albany Medical College	13
Taipei Medical University	13
University of São Paulo	12
Nanjing Medical University	10
Seoul National University	10
University of Porto	10
Anhui Medical University	9
China Medical University	9
Chinese Academy of Sciences	9
Huazhong University of Science and Technology	9
Johns Hopkins University	9
Shandong First Medical University	9
Zhejiang University	9

### 3.2 Visual analysis of published journals

To investigate the publication characteristics of journals in the fields of TH and NDs, the Bibliometrix package in R was employed for data analysis, while bubble charts were produced using ggplot2 to illustrate the publication and citation distributions across journals. Using VOSviewer (version 1.6.17), we performed a journal co-citation analysis to illustrate the interrelationships and relative influence of journals within the field. For the sake of accuracy, the journal impact factors (IFs) applied in this analysis were extracted from the 2024 edition of the Journal Citation Reports (JCR).

**Figure 3:**
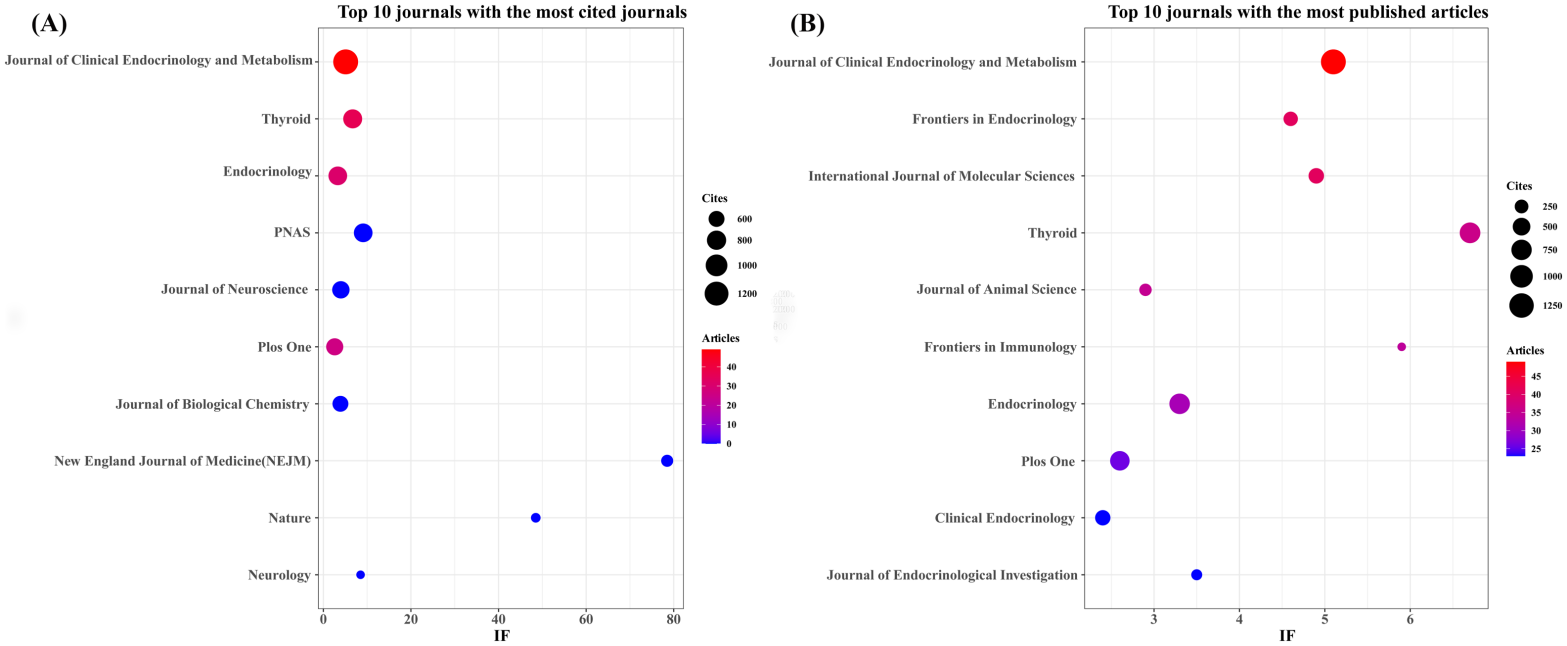
The journal with the largest number of articles published and the journal with the largest number of citations. **(A)** The journals with the highest count of citations. **(B)** The journal with the highest count of published documents.

As illustrated in Table 3 and Figure 3A, the *Journal of Clinical Endocrinology and Metabolism* ranked first in citation frequency among relevant studies (*n =* 1,310, IF = 5.1), followed by *Thyroid* (*n =* 780, IF = 6.7), *Endocrinology* (*n =* 760, IF = 3.3), *PNAS* (*n =* 760, IF = 9.1), and the *Journal of Neuroscience* (*n =* 676, IF = 4.0). Table 4 and Figure 3B indicate that The *Journal of Clinical Endocrinology and Metabolism* (*n =* 49, IF = 5.1) is the top-publishing journal in this field, followed by *Frontiers in Endocrinology* (*n =* 41, IF = 4.6), *International Journal of Molecular Sciences* (*n =* 41, IF = 4.9), *Thyroid* (*n =* 36, IF = 6.7), and the *Journal of Animal Science* (*n =* 35, IF = 2.9). These findings suggest that although several influential journals have contributed to the dissemination of research on TH and NDs, the number of publications in top-tier journals remains relatively limited. Furthermore, the overall citation quality indicates that the depth and breadth of research in this field still require further improvement.

**Table 3:** Top 10 journals with the most published articles.

Sources	Cites	Articles	IF
Journal of Clinical Endocrinology and Metabolism	1,310	49	5.1
Thyroid	780	36	6.7
Endocrinology	760	31	3.3
PNAS	760	0	9.1
Journal Of Neuroscience	676	0	4
Plos One	661	26	2.6
Journal of Biological Chemistry	598	0	3.9
New England Journal of Medicine(NEJM)	459	0	78.5
Nature	422	0	48.5
Neurology	418	0	8.5

**Table 4:** Top 10 journals with the most cited journals.

Sources	Articles	Cites	IF
Journal of Clinical Endocrinology and Metabolism	49	1,310	5.1
Frontiers in Endocrinology	41	284	4.6
International Journal of Molecular Sciences	41	344	4.9
Thyroid	36	780	6.7
Journal of Animal Science	35	197	2.9
Frontiers in Immunology	34	132	5.9
Endocrinology	31	760	3.3
Plos One	26	661	2.6
Clinical Endocrinology	23	334	2.4
Journal of Endocrinological Investigation	23	158	3.5

**Figure 4:**
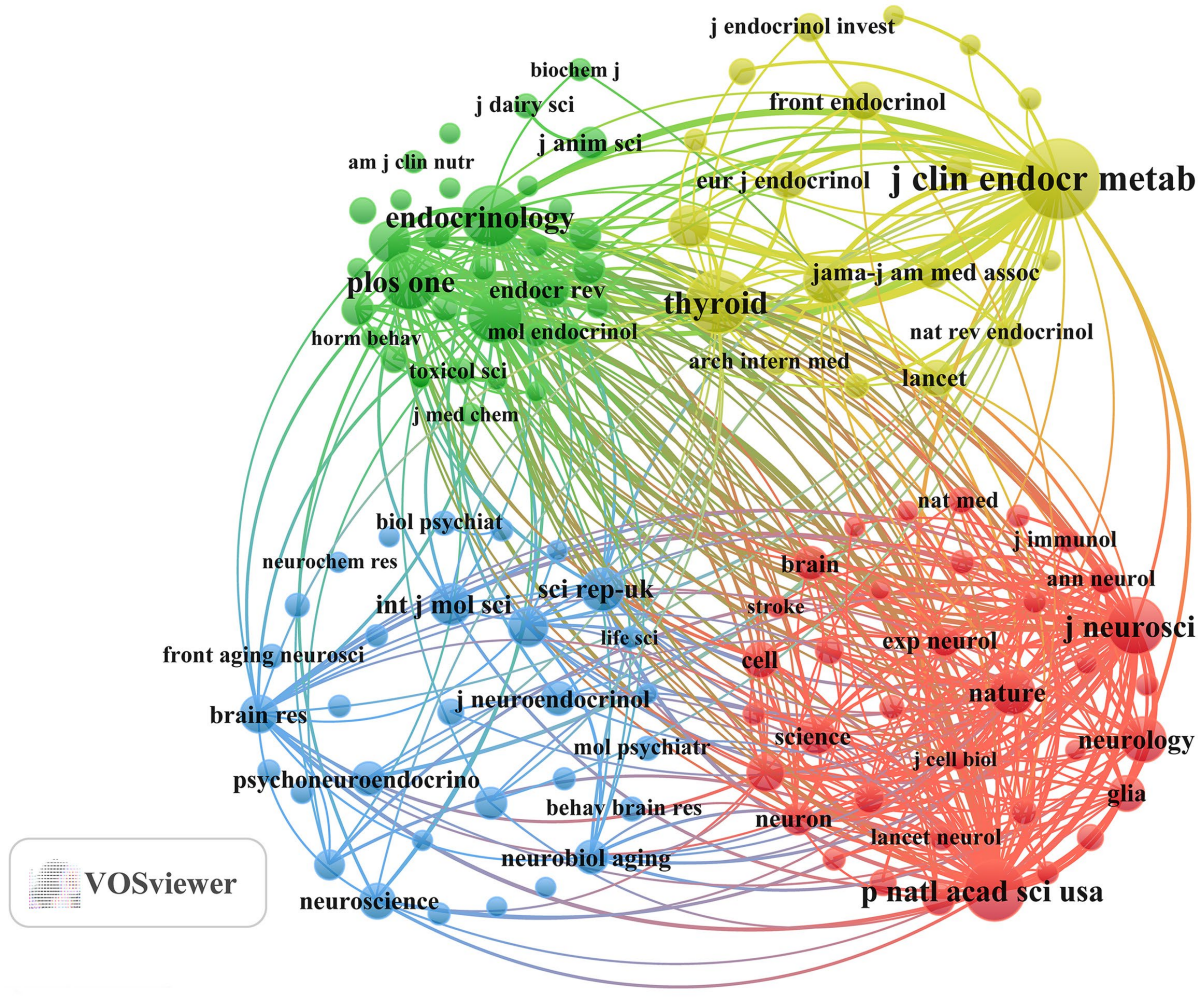
Co-cited journals involved in the relationship between TH and NDs.

The journal co-citation network diagram (Figure 4) shows that the *Journal of Clinical Endocrinology and Metabolism*, *Endocrinology*, *PNAS*, *Journal of Neuroscience*, and *Nature* are the core journals in this research field. This pattern reflects the close academic integration of neuroscience and endocrinology, as well as the important supporting role of high-quality basic research in promoting scholarly communication and development within this domain.

### 3.3 Highly cited papers and citation bursts

The bibliometrix package is an efficient tool for analyzing highly cited papers and detecting citation bursts. Using this analytical tool, the 25 most highly cited studies related to TH and NDs were identified for further analysis. Each of these papers received more than 100 citations and was distributed across 19 different journals (Table 5). The *Journal of Clinical Endocrinology and Metabolism* published the largest number of highly cited papers (*n =* 3). This journal primarily focuses on clinical endocrinology and metabolic disorders. Although it holds a certain level of influence in the TH and NDs research domain, its contribution remains limited, as no single journal exhibited a distinct dominance in publication volume. The top three most-cited papers are “Blood-Brain Barrier: From Physiology to Disease and Back” (Sweeney et al., 2019), “Establishment and Dysfunction of the Blood-Brain Barrier” (Zhao et al., 2015), and “Subclinical Hypothyroidism: A Review” (Biondi et al., 2019). All three are review articles that mainly discuss the relationship between BBB disruption and the pathogenesis of NDs, as well as the potential effects of TH deficiency on cognitive function.

**Table 5:** Top 20 cited references related to the relationship between TH and NDs.

Paper	DOI	Total citations	TC per year
Sweeney, 2019, PHYSIOL REV	10.1152/physrev.00050.2017	1,509	215.57
Zhao, 2015, CELL	10.1016/j.cell.2015.10.067	1,240	112.73
Biondi, 2019, JAMA-J AM MED ASSOC	10.1001/jama.2019.9052	392	56.00
Ravussin, 2015, J GERONTOL A-BIOL	10.1093/gerona/glv057	338	30.73
Figus, 2021, AUTOIMMUN REV	10.1016/j.autrev.2021.102776	324	64.80
Maqbool, 2016, LIFE SCI	10.1016/j.lfs.2015.10.022	251	25.10
Delivanis, 2017, J CLIN ENDOCR METAB	10.1210/jc.2017-00448	214	23.78
Liu, 2015, ENDOCRINOLOGY	10.1210/en.2014-1598	204	18.55
Genchi, 2023, INT J MOL SCI	10.3390/ijms24032633	201	67.00
De Filette, 2016, J CLIN ENDOCR METAB	10.1210/jc.2016-2300	181	18.10
Pessah, 2019, ACTA NEUROPATHOL	10.1007/s00401-019-01978-1	139	19.86
Kotwal, 2020, THYROID	10.1089/thy.2019.0250	135	22.50
Thompson, 2015, ADV MATER	10.1002/adma.201500411	135	12.27
Velasco, 2018, NUTRIENTS	10.3390/nu10030290	129	16.13
Arneson, 2018, NAT COMMUN	10.1038/s41467-018-06222-0	124	15.50
Yamauchi I, 2017, THYROID	10.1089/thy.2016.0562	119	13.22
Banks, 2019, NAT REV ENDOCRINOL	10.1038/s41574-019-0213-7	116	16.57
Orlov, 2015, J CLIN ENDOCR METAB	10.1210/jc.2014-4560	113	10.27
Chen, 2018, SCI REP-UK	10.1038/s41598-018-32886-1	112	14.00
Piketty, 2017, CLIN CHEM LAB MED	10.1515/cclm-2016-1183	107	11.89
Kojetin, 2015, NAT COMMUN	10.1038/ncomms9013	106	9.64
Li, 2017, JAMA-J AM MED ASSOC	10.1001/jama.2017.13705	104	11.56
Ruszkiewicz, 2017, J TOXICOL ENV HEAL B	10.1080/10937404.2017.1281181	103	11.44
Gereben, 2015, NAT REV ENDOCRINOL	10.1038/nrendo.2015.155	102	9.27
Li, 2019, J CELL PHYSIOL	10.1002/jcp.27180	100	14.29

**Figure 5:**
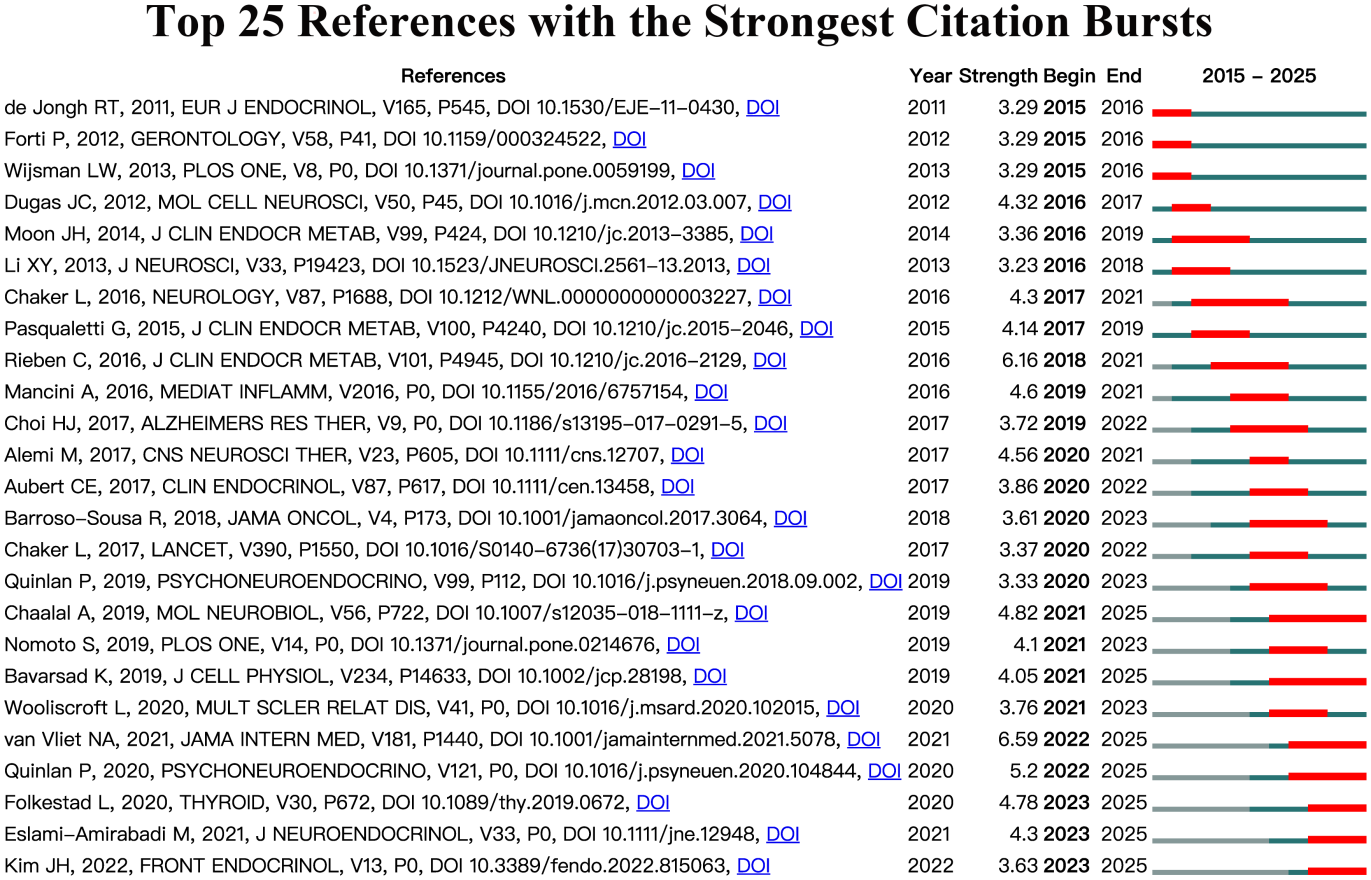
Top 25 references with the strongest citation bursts on the relationship between TH and NDs.

To explore the research frontiers and hotspots of TH and NDs in depth, this analysis utilized the CiteSpace tool to establish specific criteria (top 25; status count: 2; minimum duration: 2; slice: 1) to identify 25 papers that exhibited citation explosion phenomena (Figure 5). The titles, DOIs, and other relevant details of these papers are summarized in Supplementary material 3. Notably, the three papers with the strongest citation bursts were “Association of Thyroid Dysfunction With Cognitive Function: An Individual Participant Data Analysis” (van Vliet et al., 2021) (strength: 6.59), “Subclinical Thyroid Dysfunction and the Risk of Cognitive Decline: A Meta-Analysis of Prospective Cohort Studies” (Rieben et al., 2016) (strength: 6.16), and “Altered Thyroid Hormone Profile in Patients With Alzheimer’s Disease” (Quinlan et al., 2020) (strength: 5.20). These studies have drawn considerable academic attention for elucidating the links between thyroid dysfunction and cognitive impairment. In recent years, several emerging studies have further advanced this research frontier. The most cutting-edge works include “Graves’ Disease and Toxic Nodular Goiter, Aggravated by Duration of Hyperthyroidism, Are Associated With Alzheimer’s and Vascular Dementia: A Registry-Based Long-Term Follow-Up of Two Large Cohorts” (Folkestad et al., 2020), “Relation Between Thyroid Dysregulation and Impaired Cognition/Behavior: An Integrative Review” (Eslami-Amirabadi and Sajjadi, 2021), and “The Association Between Thyroid Diseases and Alzheimer’s Disease in a National Health Screening Cohort in Korea” (Kim et al., 2022).

Based on the analysis of highly cited papers and citation bursts, two major research directions in this field warrant particular attention. (1) Epidemiological investigations exploring the association between abnormal thyroid hormone secretion and cognitive impairment, especially the potential links between subclinical hypothyroidism (sHT), subclinical hyperthyroidism (sH), and AD. (2) Mechanistic studies focusing on the role of thyroid hormones in neurodegenerative diseases, including their regulatory effects on amyloid-*β* (Aβ) deposition, neuroinflammation, and oxidative stress, as well as their influence on myelin regeneration and neural repair.

### 3.4 Keyword clusters and evolution

Keyword clustering analysis helps researchers quickly identify the major research hotspots and developmental trends within a specific field. Using VOSviewer a total of 194 keywords were extracted among which 20 keywords appeared more than 33 times (Table 6). Keyword analysis revealed that hypothyroidism was the most common term (*n =* 128) with risk (*n =* 79) expression (*n =* 78) brain (*n =* 76) and dementia (*n =* 70) occurring frequently thereafter

**Table 6:** Top 20 keywords related to the relationship between TH and NDs.

Rank	Words	Occurrences
1	hypothyroidism	128
2	risk	79
3	expression	78
4	brain	76
5	dementia	70
6	disease	64
7	hyperthyroidism	61
8	hormone	56
9	rat	55
10	association	51
11	oxidative stress	49
12	thyrotropin	47
13	subclinical hypothyroidism	44
14	dysfunction	40
15	hippocampus	37
16	gene-expression	36
17	inflammation	36
18	thyroid function	36
19	receptor	35
20	memory	33

**Figure 6:**
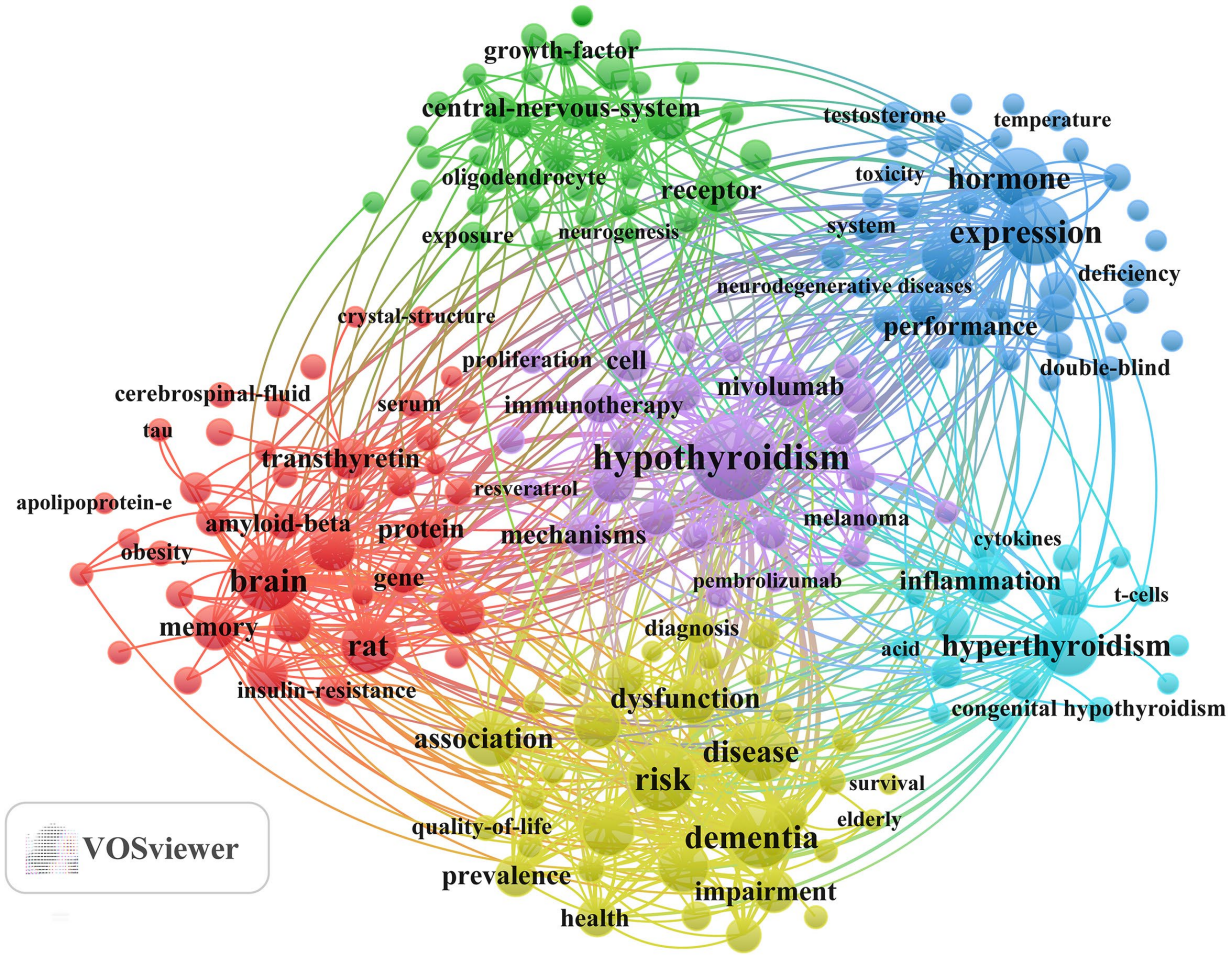
Keyword co-occurrence map of publications on the relationship between TH and NDs.

Based on a minimum occurrence threshold of 18, a total of 194 keywords were extracted for analysis. Through keyword clustering using VOSviewer, six distinct clusters were identified, each represented by a different color (Figure 6). (1) Molecular mechanisms of thyroid hormone regulation in neurodegenerative diseases (red cluster): includes 41 keywords such as brain, rat, hippocampus, gene expression, and memory. (2) Mechanisms of thyroid hormone–mediated remyelination and neural regeneration (green cluster): includes 37 keywords such as receptor, central nervous system, remyelination, differentiation, and *in vitro*. (3) Thyroid hormone regulation of energy metabolism, oxidative stress, and trace elements in neurodegenerative diseases (blue cluster): includes 36 keywords such as expression, hormone, oxidative stress, performance, and metabolism. (4) Epidemiological evidence linking thyroid dysfunction to neurodegenerative diseases (yellow cluster): includes 36 keywords such as risk, dementia, disease, association, and thyrotropin. (5) Mechanisms and therapeutic interventions for thyroid dysfunction related to immune checkpoint inhibitors (purple cluster): includes 26 keywords such as hypothyroidism, levothyroxine, nivolumab, mechanisms, and cell. (6) Inflammatory mechanisms of thyroid autoimmune diseases and the impact of maternal thyroid dysfunction on neurodevelopment (cyan cluster): includes 18 keywords such as hyperthyroidism, inflammation, Graves’ disease, model, and children. A detailed list of the keywords corresponding to the six clusters can be found in Supplementary material 3. Similarly, keyword clustering analysis of the Scopus dataset using VOSviewer identified 226 keywords, forming four distinct clusters (Supplementary Figure 2). (1) Clinical diagnosis and prognosis of endocrine adverse reactions induced by immune checkpoint inhibitors. (2) Molecular mechanisms of thyroid hormone action in neurodegenerative diseases. (3) Imaging approaches for studying the comorbidity of metabolic disorders with cognitive dysfunction and depression. (4) Immune indicators and imaging diagnostics of thyroid autoimmune diseases.

**Figure 7:**
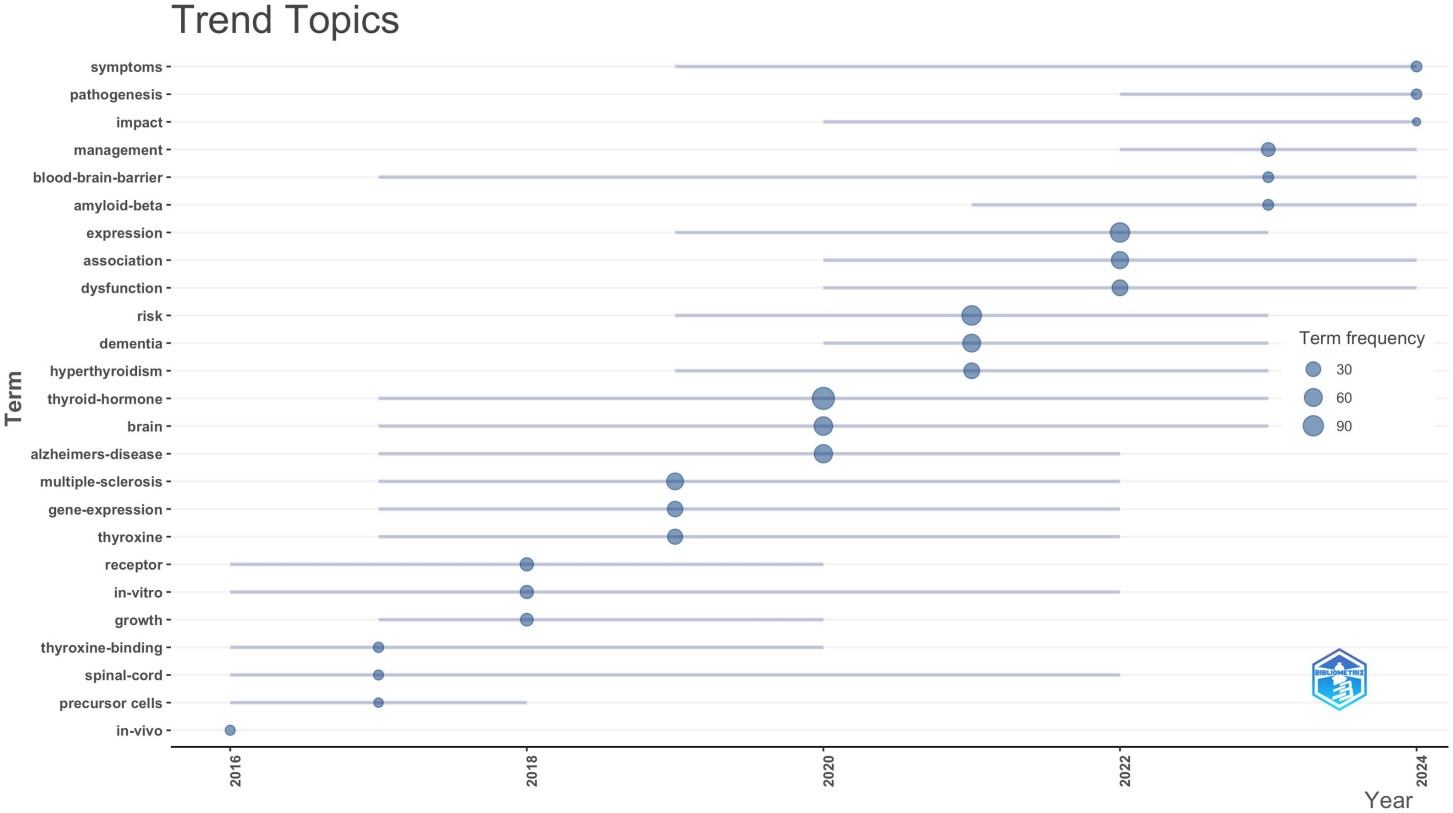
Trend topics on the relationship between TH and NDs.

To further understand the evolution of research themes in the field of TH and NDs over time, using the bibliometrix package in R, we constructed trend topic maps, which are presented in Figure 7. This visualization helps identify shifts in research focus and the progression of central themes across different temporal phases. Over the period spanning 2015 to 2017, scholarly attention was largely directed toward uncovering the basic mechanisms through which TH regulates neurodevelopment, synaptic plasticity, and myelinogenesis. Using animal and cell models, studies explored the regulatory roles of TH in these processes. Between 2018 and 2019, attention shifted toward the relationship between hormonal fluctuations and neurofunctional disorders, marking a transition from basic metabolic regulation to the investigation of immune-mediated neuroinjuries and the neuroprotective potential of TH in conditions such as multiple sclerosis and other demyelinating diseases. During 2020–2022, the focus expanded to clinical correlations between thyroid dysfunction and NDs, emphasizing the epidemiological associations between thyroid abnormalities and cognitive decline, dementia, and related disorders. From 2023 to 2025, the research emphasis evolved toward the integration of clinical management with pathological mechanisms, highlighting emerging topics such as amyloid-*β* and the BBB. This phase reflects a deepening exploration of how TH influences amyloid deposition, neuroinflammation, and BBB integrity, demonstrating the growing intersection of molecular pathology and neuroimaging. Overall, the longitudinal evolution of research themes reveals a clear trend: the field has progressively integrated fundamental biological research with clinical application, aiming to translate discoveries in endocrine regulation into effective preventive and therapeutic strategies for neurodegenerative diseases.

### 3.5 Hotspot analysis

The role of TH in NDs has attracted growing academic attention in recent years. This study conducted a comprehensive exploration of potential research hotspots through the integration of citation burst detection, keyword frequency and clustering analyses, and topic evolution analysis. Firstly, the abnormal secretion of TH and the epidemiological evidence of NDs, including abnormal hormones such as TSH and FT4, their association with AD and PD. Secondly, the mechanism research of TH in NDs, including mechanisms such as Aβ deposition, Tau phosphorylation, neuroinflammation, and oxidative stress. Finally, the role of thyroid hormone-mediated remyelination and neural regeneration mechanisms in diseases such as MS.

## 4 Discussion

### 4.1 General information

To explore the research hotspots and developmental trends concerning TH and NDs, this study carried out an integrated bibliometric and data visualization analysis based on literature retrieved from two authoritative databases: WoSCC and Scopus. Between 2015 and 2025, a total of 820 relevant articles were identified in WoSCC and 2,039 in Scopus. The annual publication trends in both databases were consistent, showing a steady upward trajectory that reflects the growing academic interest in this area. From 2020 to 2022, the number of publications increased sharply, possibly due to heightened attention to the relationship between TH and NDs during the COVID-19 pandemic. After the pandemic, the publication rate stabilized, suggesting that the field had entered a phase of sustained research activity. Among contributing countries, China emerged as the leading research contributor, ranking first with 186 publications and occupying 8 of the top 15 publishing institutions. China’s prominent position may be attributed to both its rapidly aging population and its large demographic base, which together contribute to an increasing prevalence of NDs and consequently greater scientific and clinical interest. This demographic reality has likely driven significant research investment and academic exploration in the field. The *Journal of Clinical Endocrinology and Metabolism* ranked highest among journals in both the number of publications and total citations. However, most mainstream journals in this domain currently exhibit moderate impact factors, indicating that the mechanistic depth and theoretical exploration of TH–ND research remain insufficient. Future studies should aim to address core scientific questions, deepen mechanistic investigations, and strive to publish in higher-impact journals to enhance the academic influence and international visibility of this research field.

### 4.2 Research hotspots and development trends

Through a combination of several bibliometric techniques, such as literature clustering, keyword frequency and co-occurrence analyses, and topic evolution analysis, three primary research hotspots were identified in studies concerning TH and NDs. (1) the abnormal secretion of TH and its epidemiological associations with NDs. (2) the molecular mechanisms mediating the effects of TH in the pathogenesis of NDs. (3) The contribution of TH-regulated remyelination and neural regeneration processes to the pathophysiology of demyelinating diseases, notably MS.

#### 4.2.1 Abnormal secretion of TH and epidemiological evidence of NDs

In recent years, there has been a growing body of research examining the association between TH and NDs. The results of keyword clustering and topic evolution analyses indicate that the epidemiological association between abnormal TH secretion and NDs has become a major research focus in this field.

The association between TH imbalance and AD has always been a controversial topic in the academic community. Studies have shown that higher circulating FT4 levels are an independent predictor of dementia in elderly men (Yeap et al., 2012). George et al. (2019) also pointed out that elevated FT4 levels are associated with an increased likelihood of dementia and AD. Even in cases of normal thyroid function, higher serum TSH and lower serum FT4 levels are associated with brain structural changes related to AD (Choi et al., 2017). However, the latest research presents an opposite view, refuting the relationship between high normal TSH/low normal FT4 and the risk of cognitive decline (Dori et al., 2025a). The research results question the role of TH supplementation therapy in cognitive dysfunction (Dori et al., 2025b). Additionally, the epidemiological evidence between thyroid diseases and AD also shows certain differences. Kim et al. (2022) concluded from a cohort study that significant correlations existed between AD and thyroid-related disorders, including clinical hypothyroidism, thyroiditis, and hyperthyroidism. Another large-scale study of 74,000 people argued that cognitive function was not significantly affected by subclinical hypothyroidism or hyperthyroidism (van Vliet et al., 2021). Thus, the current research results on the relationship between TH and AD are not yet completely consistent, possibly due to differences in research design, sample structure, and control of confounding factors. Despite evidence from several observational studies indicating a link between thyroid dysfunction and PD, the results have been inconsistent, and a definitive conclusion has not yet been established. In addition to AD, several studies have analyzed and demonstrated significant associations between thyroid dysfunctions—specifically hypothyroidism and hyperthyroidism—and a heightened risk of PD (Chen et al., 2020; Charoenngam et al., 2022; Wang et al., 2025). Another case–control study reached the same conclusion and emphasized that the risk is higher in women under 70 years old and men over 70 years old (Kim et al., 2021). Nevertheless, a recent bidirectional Mendelian randomization analysis reported no causal link between thyroid function and Parkinson’s disease (Zeng et al., 2024). Through the analysis of the related studies on TH and NDs, the epidemiological evidence showing the abnormal secretion of TH and NDs exhibits significant heterogeneity and uncertainty. Future research should focus on large-sample, multicenter studies to verify these observations and to explore the underlying pathological and physiological mechanisms in greater depth. These studies will help to reinforce the evidence framework and lay the theoretical groundwork for the potential use of TH in the prevention and treatment of NDs.

#### 4.2.2 Research on the mechanism of TH in NDs

In studies investigating the mechanisms of TH in neurodegenerative diseases NDs, analyses of keyword clustering and highly cited literature indicate that research has primarily focused on four key pathological processes: neuroinflammation, oxidative stress, mitochondrial dysfunction, and the phosphorylation of Tau protein and deposition of Aβ.

##### 4.2.2.1 Neuroinflammation and oxidative stress

Neuroinflammation and oxidative stress are two interrelated core pathological processes that mutually amplify each other in the progression of NDs. In AD models, the release of inflammatory cytokines exacerbates neuronal damage, while oxidative stress promotes Aβ deposition and excessive Tau phosphorylation, resulting in synaptic impairment and subsequent neuronal degeneration, thereby contributing to AD pathogenesis (Chen and Zhong, 2014). In PD, oxidative stress is recognized as a central pathogenic mechanism, whereas neuroinflammation exhibits dual roles of neuroprotection and neurotoxicity. On the one hand, neuroinflammatory activity in the central nervous system (CNS) has been shown to worsen neuronal injury (Forloni et al., 2021); on the other hand, certain microglia-mediated inflammatory reactions exert neuroprotective effects by facilitating the clearance of damaged neurons and debris (Chen and Trapp, 2016). In HD, both central and peripheral inflammatory factors are elevated, while the expression of mutant huntingtin (mHtt) further induces chronic neuroinflammation. Thyroid dysfunction, particularly hypothyroidism, has been shown to induce neuroinflammation through multiple molecular pathways. For instance, hypothyroidism can cause deletion of the RASD2 gene, which encodes a TH-regulated protein, thereby triggering neuroinflammatory responses and promoting PD pathogenesis (Napolitano et al., 2018). It can also alter toll-like receptor (TLR) signaling, activate NF-κB, and subsequently upregulate the NLRP3 inflammasome and p38 MAPK, initiating an inflammatory cascade (Bortolotto et al., 2025). Furthermore, hypothyroidism—along with oxidative stress and other factors—contributes to dopaminergic neuronal loss. Thyroid hormone supplementation has demonstrated neuroprotective potential in counteracting these effects. T3 exerts neuroprotective effects by mitigating neuroinflammation and oxidative stress, primarily via reducing reactive oxygen species (ROS) production, preventing neuronal apoptosis, and promoting synaptic function. Experimental evidence indicates that T3 supplementation in hypothyroid rats reverses memory deficits and restores key markers associated with neuroinflammation, Aβ pathology, synaptic plasticity, and memory signaling pathways (Chaalal et al., 2019). Similarly, in PD models, the neuroprotective effect of TH is attributed to its ability to inhibit neuroinflammatory signaling, reduce oxidative damage, and promote neuronal survival (Zhang et al., 2026). In addition to classic THs, thyroid hormone derivatives such as 3-iodothyronamine (T1AM) exert anti-inflammatory effects via the trace amine-associated receptor 1 (TAAR1). T1AM reduces the production of pro-inflammatory mediators—iincluding IL-6, TNF-*α*, NF-κB, MCP-1, and MIP-1, whereas it facilitates the production of the anti-inflammatory cytokine IL-10, thereby achieving a dual regulatory effect on neuroinflammation (Polini et al., 2023). THs also display antioxidant and cytoprotective properties under oxidative stress conditions (de Castro et al., 2014). Interestingly, both hyperthyroidism and hypothyroidism can disturb redox homeostasis: hyperthyroidism enhances ROS generation, whereas hypothyroidism diminishes the body’s antioxidant defense capacity (Mancini et al., 2016). These results emphasize the significance of maintaining TH homeostasis for sustaining oxidative equilibrium and reducing neurodegenerative damage.

##### 4.2.2.2 Mitochondria dysfunction

Mitochondria are organelles within eukaryotic cells that provide ATP for energy. The physiological functions of the nervous system require energy support. Mitochondrial dysfunction and insufficient energy supply may become the causes of neurodegenerative diseases (Tassone et al., 2023). Mitochondrial dysfunction is an important pathogenic mechanism of PD. HD, AD, and PD, these three neurodegenerative diseases, are all associated with the physiological process of aging, and the common mechanism is related to the reduction of mitochondrial bioenergetic efficiency (Li et al., 2021). Therefore, most animal models have verified that mitochondria play a key role in neurodegenerative diseases. TH treatment is a potential strategy to improve mitochondrial dysfunction. In the AD mouse model, it was found that T3 treatment alleviated mitochondrial dysfunction in AD by down-regulating the expression of GSK3β kinase (Maglione et al., 2022b). At the same time, T3 can activate the PINK1-parkin signaling pathway and enhance mitochondrial autophagy, thereby promoting the recovery of mitochondrial function (Chang et al., 2022). Moreover, studies have proved that TH also has the protective effect of reducing oxidative stress and improving mitochondrial function in human fibroblast cell lines.

##### 4.2.2.3 Phosphorylation of tau protein and aβ deposition

AD is characterized by two major pathological features: excessive phosphorylation of tau protein resulting in neurofibrillary tangle formation, and extracellular Aβ plaques in the brain (Li and Liu, 2024). The dysregulation of TH can significantly affect these pathological processes (Li et al., 2020). Studies have shown that the inhibition of TH signaling can exacerbate the pathological process of AD. In rat and mouse models, the inhibition of TH signaling leads to the transcriptional upregulation of the APP gene, thereby promoting Aβ production and intensifying Tau protein phosphorylation (Figueroa et al., 2021). Hypothyroidism leads to increased Aβ deposition in the hippocampus and Tau phosphorylation disorder. In contrast, normal TH signaling shows a protective effect. TH reduces APP gene expression by lowering histone H3 acetylation and lysine 4 methylation, thereby reducing Aβ formation (Belakavadi et al., 2011). Additionally, TH can enhance insulin signaling in the hippocampus, reduce pathological activation of Tau protein, and slow down the neurodegenerative process (Prieto-Almeida et al., 2018). Abnormal elevation of TH levels may also be involved in the pathology of AD. Hyperthyroid patients have significantly higher serum total Tau levels (Tan et al., 2022). Hyperthyroidism can induce brain tissue necrosis through the RIPK3/MLKL pathway, increase Aβ plaque deposition, and aggravate cognitive impairment in mice (Lou et al., 2023). Hyperactivation of the TH signaling system could promote abnormal cerebral Aβ deposition by increasing neuroserotonin levels, which act as a fibrinolytic activator inhibitor linked to histone H3 regulation.

Although this study has summarized and analyzed the key mechanisms of thyroid hormones in neurodegenerative diseases, which constitutes one of the primary research hotspots in this field, there are still obvious limitations in current research. A substantial body of research has limited its scope to examining alterations in TH levels and certain pathological markers (e.g., Aβ and inflammatory mediators) at a superficial level. The interactions between oxidative stress and the inflammatory response constitute a critical link in TH-related neurodegenerative processes, as well as the specific signaling pathways regulated by TH signaling, still lack in-depth molecular mechanistic explanations. At the same time, existing mechanistic studies, whether based on animal models of hyperthyroidism/hypothyroidism or clinical epidemiological evidence, lack strong supporting evidence. Hence, future studies should aim to dissect the complex interactions among these mechanisms and uncover the specific molecular pathways involved. This will advance mechanistic understanding, support the shift from basic research to clinical translation, and promote the therapeutic application of TH in NDs.

#### 4.2.3 The mechanism of thyroid hormone-mediated remyelination and nerve regeneration in NDs

By analyzing published articles in this field, it can be seen that the mechanism of TH–mediated remyelination and nerve regeneration is an emerging hotspot in research on TH and NDs, particularly focusing on white matter–related lesions such as MS. TH is a key regulatory factor for neural development and maturation, playing a significant role in dendrite formation, axon growth, myelinogenesis, and remyelination (Lei and Lin, 2024). TH affects the structure and function of myelin by regulating the synthesis of related proteins—myelin basic protein (MBP), proteolipid protein (PLP), and myelin-associated glycoprotein (MAG)—and the activity of associated signaling pathways.

MS is a type of white matter demyelinating lesion in the CNS (Zhang et al., 2026). One of the key focuses in MS treatment is remyelination, which not only repairs myelin lesions but is also considered a crucial mechanism for delaying or reversing the disease course (Amin and Hersh, 2021). Multiple studies using MS animal models have shown that TH promotes remyelination through mechanisms such as enhancing oligodendrocyte maturation, reducing astrocyte proliferation, regulating myelin synthesis proteins, and upregulating neurotrophic factors (Wooliscroft et al., 2020a). Additionally, oligodendrocyte precursor cells (OPCs) play a central role in remyelination by promoting neuroprotection and facilitating nerve regeneration. TH facilitate the cell cycle exit of OPCs and their differentiation into mature oligodendrocytes, thereby repairing white matter damage in MS and improving neural function (Baldassarro et al., 2022). Studies have shown that exogenous TH administration can counteract inflammation-induced remyelination disorders, enhance the regeneration process, and provide neuroprotection by partially alleviating hypothyroidism (Wooliscroft et al., 2020b). These observations offer novel therapeutic insights for NDs; however, this research direction still faces several limitations. Limitations of experimental models: Many current studies remain confined to animal models of MS, which cannot fully replicate the complexity of human NDs. This theory has not yet been sufficiently verified clinically, and there is a lack of high-quality randomized controlled trials (RCTs) to demonstrate the clinical efficacy and safety of TH supplementation for nerve regeneration and remyelination in NDs (Amin and Hersh, 2021). Unclear mechanisms: Although research in this field has progressed, the mechanism of TH-mediated remyelination and nerve regeneration is still not fully understood. Evidence indicates that TH is essential for oligodendrocyte differentiation and the process of myelinogenesis, but the specific molecular pathways remain to be further elucidated. Differences in TH effects across NDs types: Despite evidence suggesting that TH exerts a significant influence on various white matter–associated neurodegenerative disorders, its specific mechanisms differ among diseases (e.g., Alzheimer’s disease). Current research mainly focuses on MS, with relatively few studies on other NDs, and the degree of remyelination even varies among different forms of MS. In the future, more NDs models should be explored to determine whether TH-mediated remyelination and nerve regeneration follow a universal mechanism or whether similar efficacy can be observed across different types of MS.

### 4.3 Limitations

This study utilized two widely recognized databases, WoSCC and Scopus, as data sources and employed R software, VOSviewer, and CiteSpace for analysis. The bibliometric analysis delineated the principal research hotspots and emerging frontiers within the discipline of TH and NDs research. However, this study has several significant limitations.

Firstly, although this study selected data from high-quality databases that are widely used in bibliometric research, it may still have overlooked relevant literature published in other databases. Secondly, due to language restrictions, the analysis was limited to publications available in English, which might have caused the exclusion of literature published in other languages and consequently introduced potential bias into the results. Moreover, because of the restriction on article types, only reviews and research articles were selected, which might have led to the loss of some relevant data. Despite these limitations, this study still provides a relatively comprehensive perspective and a valuable reference for academic research in this field, offering meaningful guidance for future scholarly exploration.

## 5 Conclusion

This study carried out a comprehensive bibliometric analysis to explore the principal research hotspots and evolving trends in studies related to TH and NDs. The primary findings can be summarized as follows:

- Research on TH and NDs has drawn widespread international attention, led primarily by China and the United States, which also demonstrate extensive global collaboration in this domain.
- In this research field, The *Journal of Clinical Endocrinology and Metabolism* and *Frontiers in Endocrinology* are the journals that accounted for the largest share of scholarly output on this topic. The *Journal of Clinical Endocrinology and Metabolism* has established itself as a representative journal in this area due to its highest publication volume and citation frequency.
- The epidemiological association between TH secretion abnormalities and NDs has become an important research focus in this field.
- In studies on the mechanisms of TH in NDs, research has mainly focused on four pathological processes: neuroinflammation, oxidative stress, mitochondrial dysfunction, and Tau protein phosphorylation and Aβ deposition.
- The mechanism of TH–mediated remyelination and nerve regeneration has become a research hotspot in the current field of NDs treatment.

This study, using bibliometric methods, conducted a comprehensive synthesis and critical analysis of current research focuses and emerging developmental trajectories in this field. The results facilitate a quick understanding of the present research landscape and provide directions for future studies. By systematically analyzing the limitations of current research and identifying potential research frontiers, this study offers valuable insights for scholars to advance their research endeavors and foster further exploration of innovative research directions.

## Data Availability

Publicly available datasets were analyzed in this study. This data can be found at: Web of Science Scopus.
