# Supplementary material for: Global research trends on thyroid hormones and neurodegenerative diseases: a bibliometric study from 2015 to 2025
Source: Front Aging Neurosci. 2026 Apr 2;18:1780027. doi: 10.3389/fnagi.2026.1780027 (PMC13083200; doi:10.3389/fnagi.2026.1780027)
Supplement: Supplementary file 1 [file Data_Sheet_1.ZIP › Supplementary material / Supplementary material 1.docx]

wos search formula：

(TS = ("Neurodegenerative" OR "Neurodegenerative Disorder" OR "Neurologic Degenerative" OR "Alzheimer's disease" OR "Alzheimer" OR "AD" OR "Parkinson's disease" OR "PD" OR "Huntington's disease" OR "HD" OR "Amyotrophic Lateral Sclerosis" OR "ALS" OR "Spinal Cerebellar Ataxia" OR "SCA" OR "Multiple Sclerosis" OR "Cognitive impairment" OR "Cognitive decline") AND TS = ("Thyroid Hormone*" OR "Thyroid hormones" OR "Thyroxine" OR "Triiodothyronine" OR "Levothyroxine")) AND (DT = (“Article” OR “Review”)) AND (LA = (“English”)) AND (DOP = 2015-01-01/2025-09-13).

scopus search formula：

( TITLE-ABS-KEY ( "Neurodegenerative" OR "Neurodegenerative Disorder" OR "Neurologic Degenerative" OR "Alzheimer's disease" OR "Alzheimer" OR "AD" OR "Parkinson's disease" OR "PD" OR "Huntington's disease" OR "HD" OR "Amyotrophic Lateral Sclerosis" OR "ALS" OR "Spinal Cerebellar Ataxia" OR "SCA" OR "Multiple Sclerosis" OR "Cognitive impairment" OR "Cognitive decline" ) ) AND ( TITLE-ABS-KEY ("Thyroid Hormone*" OR "Thyroid hormones" OR "Thyroxine" OR "Triiodothyronine" OR "Levothyroxine") ) AND PUBYEAR > 2015 AND PUBYEAR < 2025 AND ( LIMIT-TO ( DOCTYPE , "ar" ) OR LIMIT-TO ( DOCTYPE , "re" ) ) AND ( LIMIT-TO ( LANGUAGE , "English" ) )

Results: 2039
