# Supplementary figures and images for: Global research trends on thyroid hormones and neurodegenerative diseases: a bibliometric study from 2015 to 2025
Source: Front Aging Neurosci. 2026 Apr 2;18:1780027. doi: 10.3389/fnagi.2026.1780027 (PMC13083200; doi:10.3389/fnagi.2026.1780027)

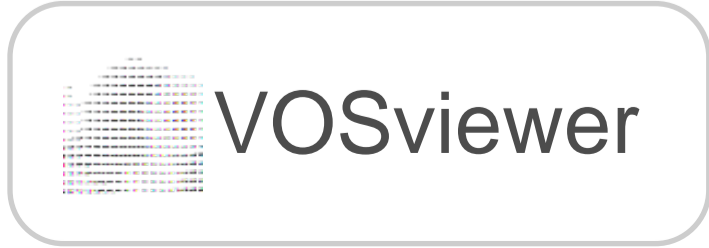

Supplement: Supplementary file 1 [file Data_Sheet_1.ZIP › Supplementary material /Supplementary Figure 2.pdf]
